# Internalizing psychological symptoms in children and adolescents with fructose malabsorption

**DOI:** 10.3389/fpsyg.2024.1414852

**Published:** 2024-07-12

**Authors:** Annabel Maurer, Adrian Lieb, Stephan Bongard

**Affiliations:** ^1^Department of Psychology, Goethe University Frankfurt, Frankfurt, Germany; ^2^Gastroenterologische Ambulanz der Klinik für Kinder- und Jugendmedizin des Universitätsklinikums Frankfurt, Frankfurt, Germany

**Keywords:** fructose malabsorption, tryptophan, serotonin, depression, anxiety, children, adolescents

## Abstract

**Introduction:**

Due to an inhibited tryptophan resorption, patients with fructose malabsorption are expected to experience decreased serotonin synthesis. A deficiency of serotonin may cause internalizing mental disorders like depression and anxiety, and a fructose-oriented eating behavior may affect these symptoms.

**Methods:**

The parents of 24 children and adolescents with a currently diagnosed fructose malabsorption aged 4;00–13;02 years (*M* = 8.10, *SD* = 2.05), the parents of 12 patients with a currently confirmed combination of fructose and lactose malabsorption aged 4;00–12;11 years (*M* = 8.07, *SD* = 2.11) and the parents of a comparative sample of 19 healthy participants aged 5;00 to 17;07 years (*M* = 9.06, *SD* = 3.04) were interviewed. The interviews were conducted using a screening questionnaire of the German “Diagnostic System of Mental Disorders in children and adolescents based on the ICD-10 and DSM-5 DISYPS-III” and a self-developed questionnaire on eating, leisure and sleeping behavior.

**Results:**

On standardized scales parents of children with fructose malabsorption reported higher levels of *Depression* compared to symptoms of *Attention-Deficit/Hyperactivity Disorders* (ADHD) and *Oppositional Defiant and Conduct Disorders* (ODD/CD). Compared to healthy controls, for patients with fructose malabsorption, higher symptom levels of *Depression* and *Anxiety* were reported. With regard to eating behavior, within the group with a combination of fructose and lactose malabsorption, a strong positive association between an increased fruit sugar consumption and higher levels of *Anxiety* and *Obsessive-Compulsive Disorders/Tics* were found.

**Discussion:**

These results suggest a close association between fructose malabsorption and elevated internalizing psychological symptoms in children and adolescents.

**Clinical trial registration:**https://drks.de/search/en/trial/DRKS00031047, DRKS-ID [DRKS00031047].

## Introduction

Fructose malabsorption is a common carbohydrate metabolism disorder. In Europe, about one third of adults and two out of three children are affected by fructose malabsorption (Kretschmer, 2022). The present study is based on the consideration that children and adolescents with an untreated fructose malabsorption may suffer from concomitant mental symptoms, which are associated with a reduced intestinal absorption of tryptophan and therefore dysregulated processes of the serotonergic system. It is generally assumed that there is an impaired tryptophan absorption in patients with fructose malabsorption (Ledochowski et al., 2001). The intestinal walls of affected individuals contain only few or impaired passive transport channels, so-called GLUT-5 transporters (Velten and Bayerl, 2007). Those are unable to react adequately to the entry of fructose. As a result, ingested fruit sugar enters virtually undigested into the large intestine, where it is metabolized—in parts—into short-chain fatty acids, hydrogen, and carbon dioxide (Ledochowski et al., 2000b). Among other symptoms, most of these metabolites can cause bloating, abdominal pain, nausea, and vomiting in individuals with fructose malabsorption (Escobar et al., 2014); constipation and diarrhea are also reported (Ledochowski et al., 2000b). Food high in fructose content, like various types of fruits and juices, as well as food enriched with fruit sugar, is described as causing the most discomfort in affected individuals (Latulippe and Skoog, 2011). While metabolized parts in the large intestine may lead to the mentioned gastroenterological complaints in individuals with a dietary fructose intolerance, remaining non-metabolized parts bind to the amino acid tryptophan. As a consequence, the essential amino acid is not available for further metabolic processes—such as the synthesis of serotonin (Huether et al., 1998)—to the same extent as in persons without fructose malabsorption (Ledochowski et al., 2001). Serotonin is known to modulate a variety of physiological processes (Berger et al., 2009) and a deficiency of serotonin can promote depressive symptoms (Mehler-Wex and Kölch, 2008; Jenkins et al., 2016). A study by Ledochowski et al. (2001) discovered lowered blood tryptophan levels in individuals with fructose malabsorption. Compared to a healthy sample, the female participants were also reported to have a higher expression of depressive symptoms. Varea et al. (2004) examined fourteen adolescents, eight of whom suffered from a combined fructose and lactose malabsorption, five from a lactose malabsorption, and one person was known to have an isolated fructose malabsorption. The results showed an association between depressive symptoms and the combined form of fructose and lactose malabsorption. A more recent study by Enko et al. (2018) revealed an association between fructose malabsorption and depressive symptoms. Other studies showed that a diet adapted to fructose malabsorption led most affected individuals to experience relief not only of physical symptoms (Litschauer-Poursadrollah et al., 2012; Berni Canani et al., 2016), but with regard to depression, also of psychological complaints (Ledochowski et al., 2000a). Based on these previous results, we hypothesized that an impaired absorption of tryptophan in children and adolescents with fructose malabsorption may promote psychological symptoms that can be associated with serotonergic processes. In addition to depressive symptoms (Ledochowski et al., 1998), special attention was given to anxiety (Deister, 2013) and obsessive-compulsive spectrum disorders (Kis et al., 2007). Because in some cases tic disorders have been reported to be associated with the serotonergic system (Steeves and Fox, 2008), they were also included in this study.

## Hypotheses

It was hypothesized that children and adolescents with fructose malabsorption more likely show heightened serotonin-associated internalizing psychological symptoms (*Anxiety* and *Depression* extended by *Obsessive-Compulsive Disorders/Tics*) than externalizing ones (*Attention-Deficit/Hyperactivity Disorders* and *Oppositional Defiant and Conduct Disorders*). Additionally, it was hypothesized that they would show higher levels of internalizing mental symptoms than a healthy control group. Also, the degree of symptom expression was assumed to be related to eating behavior. It was expected, that within the group of children and adolescents with fructose malabsorption, the quantity of fruit sugar consumption would correlate with the degree of internalizing psychological symptoms.

## Methods

### Sample

Children and adolescents and their caregivers were recruited by support of the ambulatory gastroenterological department for children and adolescents of the Goethe-University Hospital in Frankfurt am Main, Germany. A total of 43 patients (*n* = 22 female, *n =* 21 male) who had been referred to the University Hospital due to a suspected fructose malabsorption or a combination of a fructose and lactose malabsorption formed the initial sample. Patients for whom tests disconfirmed the diagnosis of a malabsorption (*n* = 7) were obtained as a control sample. This was then supplemented by 12 additionally recruited children and adolescents without confirmed fructose and/or lactose malabsorption to a total of 19 persons (16 female).

The age of the group “Fructose Malabsorption” (*n* = 24) ranged from 4;00 to 13;02 years (*M* = 8.10, *SD* = 2.05) for 9 female and 15 male participants. The age of the group “Fructose and Lactose Malabsorption combined” (*n* = 12) ranged from 4;00 to 12;11 years (*M* = 8.07, *SD* = 2.11) for 6 female and 6 male participants. The control sample ranged in age from 5;00 to 17;07 years (*M* = 9.06, *SD* = 3.04), for sixteen female and three male participants. The described differences in gender distribution in the three aforementioned groups were statistically significant [*F*(2, 54) = 5.54; *p* = 0.007], but no group differences could be secured with regard to age [*F*(2, 54) = 0.99; *p* = 0.38]. Participation was voluntary and without compensation. Families were informed in advance—both verbally and in writing—about the study and how it would be conducted and gave written informed consent to participate. Parents were able to voluntarily provide their e-mail address in order to receive more detailed information about the study results after the survey was completed. The e-mail address was stored separately from the study data.

The study was approved by the ethics committee of the Department of Psychology and Sport Sciences, Goethe University Frankfurt, Germany.

### Material

Three different questionnaires were used which were completed in their parent-rating version in paper-pencil format. They are explained in the following.

#### DISYPS-III questionnaire for the screening of mental disorders (FBB-SCREEN)

At first the *Questionnaire for the Screening of mental disorders FBB-SCREEN*, a screening questionnaire of the German *Diagnostic System of Mental Disorders in children and adolescents based on the ICD-10 and DSM-5 DISYPS-III* (Döpfner and Görtz-Dorten, 2017) was filled out by the parents. The *DISYPS-III* is a diagnostic system that covers mental disorders in children and adolescents aged 4;00–17;11 years. The used *FFB-SCREEN* captures a broad spectrum of psychological symptoms in an economical way and provides results that can justify the administration of further disorder-specific instruments. With 49 items it records initial indications of the presence of mental disorders related to *ICD-10* and *DSM-5*. Items are answered on a four-point Likert scale, on which symptom severity is rated from 0 to 3. The majority of items can be subsumed to the scales *Screening Attention-Deficit/Hyperactivity Disorders (ADHD)*, *Screening Oppositional Defiant and Conduct Disorders (ODD/CD)*, *Screening Anxiety*, *Screening Depression*, *Screening Developmental and Excretory Disorders*, *Screening Autism* and *Screening Obsessive-Compulsive Disorders/Tics (OCD/Tics)*. Except for the scales S*creening Developmental and Excretory Disorders* and *Screening Obsessive-Compulsive Disorders/Tics*, sufficient internal consistencies between α = 0.67 and α = 0.92 are reported. For the two scales with less satisfactory internal consistencies, the scales’ authors recommend further data analysis on item level.

These so-called subscales can largely be assigned to the following superordinate scales: *Screening External* (*Screening ADHD*, *Screening ODD/CD*), *Screening Internal* (*Screening Anxiety*, *Screening Depression*), *Screening Contact Behavior* (*Screening Autism* plus items related to *Social Anxiety*, *Mutism* and *Reactive Attachment Disorder*), which all show internal consistencies of > 0.80. The 49 items can also be combined to calculate a total score for overall symptoms (α = 0.92). For parent reports, representative norms are available for girls and boys in the age ranges 4–6;11 years, 7–10;11 years, 11–13;11 years and 14–17;11 years.

#### Questionnaire on eating, leisure and sleeping behavior

The questionnaire on eating, leisure and sleeping behavior was specifically designed for this study. Thus, information on its psychometric properties is not yet available. With regard to eating habits, this questionnaire primarily records what the child usually eats and drinks at breakfast and during school break. In addition, questions are asked about snacks and beverages over the course of the day. All questions have different answer options that can be rated by parents using a four-point Likert scale from “never,” “rarely,” “frequently” to “always.” Additionally, it is requested how often the child asks for sweets on school days or weekends and how often he or she is actually allowed to eat them. Here the assessments are also made on four-point scales from “not at all,” “once,” “twice” to “three times and more” per day. The data on leisure time and sleep behavior was not analyzed in the context of this study, all related information will be presented elsewhere.

#### Type of malabsorption

Finally, an also self-developed five-item-questionnaire for recording the type of malabsorption was used. It documented which form of malabsorption has been confirmed or excluded at the current appointment. Additionally, it asked whether a malabsorption has been diagnosed or disconfirmed at an earlier point in time and whether further test appointments are planned. It hereby assigned the subjects to the group “Fructose Malabsorption,” “Fructose and Lactose Malabsorption combined” or “Control Sample.”

### Procedure

A hydrogen breath test was used as diagnostic tool to confirm the presence of fructose or lactose malabsorption. In this test, after the administration of a liquid fructose or lactose concentrate adjusted to the patient’s weight, the hydrogen content of breath is measured every 30 min over a period of at least 120 min. Malabsorption is considered confirmed as far as the concentration of hydrogen rises to a critical value or significantly exceeds the baseline value (Jones et al., 2011).

The questionnaires of the study sample were completed by participants’ parents during their waiting time before and between examinations in the ambulatory gastroenterological department in following order: first, the *FFB-SCREEN*, then the questionnaire on eating, leisure and sleeping behavior and finally the questionnaire on type of malabsorption(s). To the caregivers of the later recruited control sample (*n* = 12) only the *DISYPS-III FBB-SCREEN* was handed out. The information sheet and the declaration of consent (including a duplicate to take home) for the study were provided for all participants on the first pages of a folder containing the questionnaire(s) to be completed. The folders of the study sample were handed out by the medical assistant to families whose child was suspected of suffering from fructose malabsorption. The folders of the later recruited control sample were given out to participating families by the investigator. The processing time of the study sample was about 30 min and that of the supplementary control sample took approximately 15 min.

### Data processing and data analyses

Incomplete sheets of the *DISYPS-III FBB-SCREEN* were excluded from data analysis. Statistical analysis of this non-experimental questionnaire study was mainly performed by using SPSS 25.0 (Chicago, United States). First, data was tested for normal distribution using the Shapiro–Wilk-test. The analyses revealed that within the groups not all dependent variables could be assumed to be normally distributed. Therefore, the further inferential statistical analyses were performed non-parametrically. Post-hoc Holm-Bonferroni correction was carried out with an independent calculator to adjust the α-level (Hemmerich, 2016), as SPSS does not offer this procedure.

All scale-related data presented in the following refer to the screening scales of the *DISYPS-III FBB-SCREEN*, even if this is no longer explicitly stated for the sake of better readability.

Within the *DISYPS-III FBB-SCREEN*, stanine values higher than or equal to eight are considered as clinically relevant (Döpfner and Görtz-Dorten, 2017).

## Results

### Symptomatology within the group “fructose malabsorption”

With regard to the superordinate scales a preliminary descriptive examination of the “Fructose Malabsorption” group’s data (see Table 1) showed a higher mean stanine value on the scale *Internal* than on the scale *External* which subsequently proved to be statistically significant (*z* = 2.85, *p* = 0.004, Wilcoxon-test). Further inferential statistical data analysis confirmed a significant discrepancy between symptom expression on the scale *Depression* and the scores on the scales *Attention-Deficit/Hyperactivity Disorders ADHD* (*z* = 2.79, *p* = 0.005) and *Oppositional Defiant and Conduct Disorders ODD/CD* (*z* = 2.82, *p* = 0.005). All other hypothesis-relevant analyses did not reveal significant median differences (all *z* < 1.92; all *p* > 0.055). In line with these results, with a mode score of eight, a value in clinically relevant range was recorded for the scale *Depression*. Twelve out of twenty-four (50%) participants with confirmed fructose malabsorption reached stanine scores of ≥8 on the scale *Depression*, respectively a clinically relevant degree of depressive symptoms.

**Table 1:** Means (M), standard deviations (SD), medians and modes of the scales’ stanine scores for all groups in the observed scales (*N* = 55).

		“Fructose malabsorption” (*n* = 24)	“Fructose and lactose malabsorption combined” (*n* = 12)	“Control sample” (*n* = 19)
ADHD^b^	M SD	5.75 1.86	7.17 1.19	5.05 1.73
	Median Range	6.00 2.50–9.00	7.00 5.00–9.00	5.00 2.50–8.00
	Mode	5.00	7.00	5.00
ODD/CD^c^	M SD	5.44 2.04	7.00 0.85	5.66 2.04
	Median Range	5.00 2.50–9.00	7.00 6.00–8.00	6.00 2.50–8.00
	Mode	5.00	9.00^a^	6.00^a^
Anxiety	M SD	6.35 1.46	7.00 1.81	4.58 1.96
	Median Range	6.50 2.50–9.00	7.00 4.00–9.00	5.00 2.00–8.00
	Mode	7.00	9.00	6.00
Depression	M SD	7.04 1.52	7.83 0.94	5.63 1.92
	Median Range	7.50 3.00–9.00	8.00 6.00–9.00	6.00 3.00–8.00
	Mode	8.00	8.00	6.00^a^
OCD/Tics^d^	M SD	4.88 2.18	6.13 2.43	4.39 1.31
	Median Range	3.75 3.50–9.00	5.75 3.50–9.00	4.00 3.50–8.00
	Mode	3.50	9.00	4.00
External^e^	M SD	5.69 1.59	7.08 1.08	5.26 1.62
	Median Range	5.00 2.50–9.00	7.00 5.00–9.00	5.00 2.00–8.00
	Mode	5.00	7.00	5.00
Internal^f^	M SD	6.71 1.30	7.42 1.44	5.11 1.70
	Median Range	6.50 5.00–9.00	7.50 5.00–9.00	5.00 2.00–8.00
	Mode	6.00	9.00	6.00

In some cases, the *DISYPS-III* manual lists several stanine values that can be assigned to a raw score of 0. Therefore, the mean of the stanine values listed was calculated, resulting in the decimal numbers given.

a The highest mode score is presented.

b ADHD, Attention-Deficit/Hyperactivity Disorders.

c ODD/CD, Oppositional Defiant and Conduct Disorders.

d OCD/Tics, Obsessive-Compulsive Disorders/Tics.

e External: ADHD, ODD/CD.

f Internal: Anxiety, Depression.

### Symptomatology within the group “fructose and lactose malabsorption combined”

For the group “Fructose and Lactose Malabsorption combined,” descriptive statistics also showed a higher mean stanine value on the superordinate scale *Internal* than on the scale *External* (see Table 1). However, this discrepancy did not prove to be statistically significant (*z* = 0.92, *p* = 0.36). The inferential statistical analysis only confirmed a significant discrepancy between the expression on the symptom scale *Depression* compared to the scale *ODD/CD* (*z* = 2.49, *p* = 0.013, Wilcoxon-test) for the group of children and adolescents suffering from both fructose and lactose malabsorption. All other hypothesis-relevant discrepancy tests within this group did not identify significant median differences (all *z* < 1.71 all *p* > 0.088).

Considering the distribution of measurements within the group “Fructose and Lactose Malabsorption combined” according to clinically relevant modal values, it was shown that six out of twelve children achieved stanine values of ≥8 on the superordinate scale *Internal*. With further regard to the associated subscales eight out of twelve children (67%) showed a depression stanine score of ≥8 and five of twelve (42%) showed a stanine score of ≥8 on the scale *Anxiety*. On the symptom scale *OCD/Tics*, four out of twelve (33%) subjects reached a stanine value of ≥ 8. Although not hypothesized, it should be mentioned for completeness, that the distribution of stanines of the scale *Screening ODD/CD* showed that four out of twelve children (33%) reached a clinically relevant stanine value of ≥8. Overall, a striking clustering of clinically relevant scores on the hypothesis-relevant scales could be found in children and adolescents with a combination of fructose and lactose malabsorption-including the scale *Obsessive-Compulsive Disorders/Tics*.

### Pairwise comparisons of symptom expression between the groups “fructose malabsorption,” “fructose and lactose malabsorption combined” and “control sample”

Descriptive comparisons of the groups “Fructose Malabsorption” and “Control Sample” showed noticeable differences according to mean stanine scores on the superordinate scale *Internal* as well as on its subscales *Depression* and *Anxiety* with higher symptom severity observed in children and adolescents with fructose malabsorption. Within the control group, no modal value reached clinically relevant severity. The complete descriptive statistics of the control sample are presented in Table 1. Next, participants with a combined fructose and lactose malabsorption (*n* = 12) were compared to healthy controls (*n* = 19) by sight. Here, descriptive observation of the data showed elevated mean differences on almost all scales (see Table 1).

Non parametric Kruskal-Wallis Test for independent samples revealed significant group differences on nearly all observed scales (*Attention-Deficit/Hyperactivity Disorders*: *p* = 0.005, *Oppositional Defiant and Conduct Disorders*: *p* = 0.039, *Anxiety*: *p* = 0.002, *Depression*: *p* = 0.003; *External*: *p* = 0.007, *Internal*: *p* = <0.001), except *Obsessive-Compulsive Disorders/Tics* (*p* = 0.113).

Pairwise comparisons of symptom expression between the group of children and adolescents with fructose malabsorption and the control sample primary confirmed higher symptom expression on the superordinate scale *Internal* for the clinical group “Fructose Malabsorption” (see Table 2). In addition, subjects with fructose malabsorption also showed significantly higher symptom expression than participants without confirmed malabsorption on both internalizing subscales, *Depression* and *Anxiety*.

**Table 2:** Pairwise comparisons of symptom expression between all groups (*N* = 55).

		“Fructose malabsorption” **>** “Control sample”	“Fructose malabsorption” **<** “Fructose and lactose malabsorption combined”	“Fructose and lactose malabsorption combined” **>** “Control sample”
ADHD^a^	*p*	0.209	0.024*	0.001**
	*adj. p* Holm-Bonferroni	0.209	0.048*	0.003**
ODD/CD^b^	*p*	0.405	0.011*	0.082
	*adj. p* Holm-Bonferroni	0.405	0.033*	0.164
Anxiety	*p*	0.003**	0.485	0.002**
	*adj. p* Holm-Bonferroni	0.006**	0.485	0.006**
Depression	*p*	0.017*	0.160	<0.001***
	*adj. p* Holm-Bonferroni	0.034*	0.160	0.003**
OCD/Tics^c^	*p*	0.453	0.037*	0.169
	*adj. p* Holm-Bonferroni	0.453	0.111	0.338
External^d^	*p*	0.604	0.007**	0.003**
	*adj. p* Holm-Bonferroni	0.604	0.014*	0.009**
Internal^e^	*p*	0.006**	0.217	<0.001***
	*adj. p* Holm-Bonferroni	0.012*	0.217	0.003**

Asymptotic significances (2-sided tests) are displayed (*p < 0.05, **p < 0.01, ***p < 0.001). Direction of difference is mentioned above stated as “greater than/less than” signs. Significance values have been adjusted by the Holm-Bonferroni correction for multiple tests.

a ADHD, Attention-Deficit/Hyperactivity Disorders.

b ODD/CD, Oppositional Defiant and Conduct Disorders.

c OCD/Tics, Obsessive-Compulsive Disorders/Tics.

d External: ADHD, ODD/CD.

e Internal: Anxiety, Depression.

With regard to the scale *OCD/Tics* the comparison of both groups did not yield statistical significance. The supplementary statistical comparisons of the expressions on the symptom scales *Attention-Deficit/Hyperactivity Disorders* and *Oppositional Defiant and Conduct Disorders* also determined no significant group differences.

Pairwise comparison of the group “Fructose and Lactose Malabsorption combined” and the “Control Sample” also confirmed a higher symptom expression on both the superordinate scale *Internal* and on its associated subscales *Depression* and *Anxiety* for the clinical group (see Table 2). Comparisons of the aforementioned groups in terms of recorded symptom severity on the scale *External* and on its subscale *Attention-Deficit/Hyperactivity Disorders* also showed significant differences with higher symptom levels for the group with combined malabsorption. The comparison of the central tendencies of the hypothesis-relevant scale *OCD/Tics* did not reveal significant group differences. With regard to the second *External*-subscale, *ODD/CD*, the comparison between the groups “Fructose and Lactose Malabsorption combined” and “Control Sample” did not reveal a significant difference as well.

Pairwise comparison between the clinical groups “Fructose Malabsorption” and “Fructose and Lactose Malabsorption combined” revealed a significantly higher symptom expression on the scale *External* as well as on its two subscales (*ADHD*, *ODD/CD*) for the group with combined malabsorption (see Table 2).

### Fruit sugar related eating habits and symptom expression within the groups “fructose malabsorption” and “fructose and lactose malabsorption combined”

In a last set of analyses, possible relationships between eating habits and psychological symptoms were calculated via Spearman-Rho correlations (one-tailed tests). For the group “Fructose Malabsorption”, a medium-sized, positive relationship between a heightened fructose consumption and depressive symptoms was found [*r_s_*(24) = 0.35, *p* = 0.084]. The other correlations were rather low and insignificant [*Anxiety* (*r_s_*(24) = −0.176, *p* = 0.21); *OCD/Tics* (*r_s_*(24) = 0.146, *p* = 0.25); *Internal* (*r_s_*(24) = −0.071, *p* = 0.37)].

For the group, “Fructose and Lactose Malabsorption combined” (*n* = 12) a positive correlation was initially found between a heightened fructose consumption and symptom expression on the superordinate scale *Internal* [*r_s_*(12) = 0.560, *p* = 0.029]. With regard to the associated subscales, a strong positive influence of a high fructose diet on the related symptom expression could be demonstrated for the scale *Anxiety* [*r_s_*(12) = 0.620, *p* = 0.016]. Regarding the expression on the subscale *Depression* [*r_s_*(12) = 0.443, *p* = 0.074], a moderately strong but only tendentially significant correlation was found. Finally, for the group “Fructose and Lactose Malabsorption combined,” a high positive correlation between a fructose-oriented eating behavior and an increased symptom expression could be objectified on the symptom scale *Obsessive-Compulsive Disorders/Tics* [*r_s_*(12) = 0.527, *p* = 0.039].

## Discussion

The results of this study show that children and adolescents with an untreated fructose malabsorption frequently display internalizing psychological symptoms. For half of twenty-four patients a clinically relevant degree of depressive symptoms was recorded. In accordance with our initial hypothesis, children and adolescents with fructose malabsorption were reported higher symptom expression on the superordinate scale *Internal* (*Depression*, *Anxiety Disorders*) compared to the superordinate scale *External* (*Attention-Deficit/Hyperactivity Disorders*, *Oppositional Defiant and Conduct Disorders*). Compared to healthy controls, for children and adolescents with a currently diagnosed fructose malabsorption a higher symptom expression on the scale *Internal* and on its two subscales—*Anxiety* and *Depression*—was found. The presented results extend earlier results by Ledochowski et al. (1998, 2000c) and also findings by Enko et al. (2018), who mainly observed depressive symptoms in affected individuals. In accordance with Ledochowski et al. (2001) we assume that the reported internalizing psychological symptoms are likely the consequence of an impaired tryptophan absorption in affected patients, although we have not investigated this directly and thus are not able to prove it. Further research should address this assumption in more detail, although a former study by Varea et al. (2005) was not able to confirm evidence on this suggestion. Other factors that may have influenced the results should also be mentioned. For example, chronic abdominal pain as a result of carbohydrate metabolism disorder, may have an influence on the mental constitution of affected children and adolescents. Mention should also be made of a recent study where Enko et al. (2020) found a connection between Interleukin-6, tryptophan metabolism and depressive symptoms in patients with fructose malabsorption, lactose malabsorption or the combination of both. Compared to patients without signs of depression, increased serum levels of the inflammatory marker Interleukin-6 were found in patients suffering from depressive symptoms.

All this implies that further studies will be necessary to specify potential mechanisms.

After integrating all observations, it remains to be mentioned that further studies with an expanded sample size of children and adolescents with a combined fructose and lactose malabsorption also appear to be of importance. In terms of internalizing psychological symptoms within this group we only found a higher expression on the symptom scale *Depression* compared to *Oppositional Defiant and Conduct Disorders*. Nevertheless, descriptive data analyses of the group “Fructose and Lactose Malabsorption combined” revealed that for a majority (67%) of patients clinically relevant values of *Depression* were reported. For about half (42%) of the examined patients clinically relevant symptoms of *Anxiety,* and for notably one third of the sample (33%) clinically relevant symptoms of *Obsessive-Compulsive Disorders/Tics* were described. Additionally, the pairwise comparison of patients suffering from a combination of fructose and lactose malabsorption with healthy controls confirmed not only a higher expression of internalizing mental symptoms (scales *Internal*, *Depression*, *Anxiety*) but also of externalizing ones (scales *External*, *Attention-Deficit/Hyperactivity Disorders*) in the patient’s group. These observations extend previous outcomes by Varea et al. (2004, 2005) that only revealed increased depressive symptoms in children and adolescents with a combination of fructose and lactose malabsorption and may therefore lead to further studies.

Finally, for the group suffering from a combined malabsorption we were able to confirm a positive association between a fructose-rich diet and internalizing psychological symptoms. With regard to *Anxiety*, a strong influence of a fructose-rich diet on symptom severity was shown. For symptoms of *Depression* a moderate correlation was found. Relating to eating habits we finally confirmed for the group with combined malabsorption a positive correlation between a fructose-rich diet and an increased symptom expression concerning *Obsessive-Compulsive Disorders/Tics*. This has not been described previously and may also inspire further investigations.

The presented study has some limitations that need to be addressed. The small and unbalanced sample limits the generalizability. The use of a parent-rating questionnaire also represents sort of a restriction. Nevertheless, its use seemed necessary and reasonable to capture a broad spectrum of psychopathological irregularities without putting unnecessary strain on the participating children and adolescents. It was also regarded suitable to affirm initial suppositions to consider them more intensively at a later stage.

## Conclusion

The obtained results suggest that children and adolescents with an untreated fructose malabsorption or a combined form of fructose and lactose malabsorption may suffer from internalizing psychological symptoms. For children and adolescents with a combined malabsorption also the presence of possible externalizing psychological symptoms cannot be excluded at the moment. In this regard a comparison of this group with the group “Fructose Malabsorption” also needs to be mentioned, where we found significantly higher levels of externalizing psychological symptoms related to the scale *External* and its two subscales *ADHD* and *ODD/CD* in patients with combined malabsorption.

According to our actual state of knowledge, it seems to be beneficial to record the history of psychological symptoms in the context of an initial gastroenterological investigation on suspicion of malabsorption(s). At the same time, it could also be advantageous to explore the presence of possible malabsorption(s) in the setting of initial psychological examinations. Finally—and in order to be able to offer optimal treatment options to affected children and adolescents—a close cooperation between clinicians from gastroenterology and psychology should be taken into account in the future.

## Data availability statement

The raw data supporting the conclusions of this article will be made available by the authors, without undue reservation.

## Ethics statement

This study involving humans was approved by the ethics committee of the Department of Psychology and Sport Sciences, Goethe-University Frankfurt. Ethics approval number: 2017-31. The study was conducted in accordance with the local legislation and institutional requirements. Written informed consent for participation in this study was provided by the participants’ legal guardians/next of kin. Written informed consent was obtained from the minor(s)’ legal guardian/next of kin for the publication of any potentially identifiable images or data included in this article.

## Author contributions

AM: Conceptualization, Investigation, Resources, Validation, Visualization, Writing – original draft, Writing – review & editing, Data curation, Formal analysis, Methodology. AL: Investigation, Resources, Writing – review & editing, Data curation. SB: Conceptualization, Formal analysis, Investigation, Methodology, Supervision, Visualization, Writing – review & editing, Project administration.
